# Quantification of cAMP and cGMP analogs in intact cells: pitfals in enzyme immunoassays for cyclic nucleotides

**DOI:** 10.1186/1471-2210-11-S1-P75

**Published:** 2011-08-01

**Authors:** Katharina Werner, Frank Schwede, Hans-Gottfried Genieser, Jörg Geiger, Elke Butt

**Affiliations:** 1 Institute for Clinical Biochemistry and Pathobiochemistry, University of Wuerzburg, Germany; 2 Biolog Life Science Institute, Flughafendamm 9a, D-28199 Bremen, Germany

## Background

The present work evaluates the cross-reactivity of commercially available cyclic nucleotide analogs with cAMP- and cGMP-immunoassays from Cayman, IBL (both IBL International, Hamburg, Germany) and ENZO Life Sciences (Loerrach, Germany).

## Results and conclusion

Most of the tested cyclic nucleotide analogs showed low degree competition with the antibodies; however, with Rp-cAMPS, 8-Br-cGMP and 8-pCPT-cGMP a strong cross-reactivity with the ENZO cAMP- respectively cGMP-EIA and the IBL cGMP-RIA was observed (Table 1). As a consequence we tested these derivatives with the Cayman cGMP-EIA. This assay is less sensitive to cGMP (1.0 pmol/ml) than the ENZO cGMP-EIA (0.01 pmol/ml), however the specificity concerning cGMP-analogs is superior and therefore advantageous when measuring cGMP in the presence of 8-Br-cGMP or 8-pCPT-cGMP.

The determined EIA binding constants enabled the measurement of the intracellular cyclic nucleotide concentrations and revealed a time- and lipophilicity-dependent cell membrane permeability of the compounds in the range of 10-30 % of the extracellular applied concentration after 20 min (Table 1).

**Table 1:** Lipophilicity (log K_w_), cell permeability and EIA/RIA specificity of selected cyclic Nucleotide analogs.

Analog	Log K_w_	Permeability	Specificity ENZO cAMP-EIA	Specificity ENZO cAMP-EIA	Specificity IBL cGMP-RIA	Specificity Cayman cGMP-EIA
2’-dcGMP	0.65	0%		5.21%		
**cGMP**	**0.77**			**100%**	**100%**	**100%**
Rp-cGMPS	0.89			0.27%	10.6%	
2’-dcAMP		0%	2.4%			
**cAMP**	**1.09**		**100%**			
8-Br-cGMP	1.17	12.1%		490%	20%	0.5%
Rp-cAMPS	1.21	12.2%	68%			
8-Br-cAMP	1.35	8.0%	0.4%			
Rp-8-Br-cAMPS	1.47		0.3%			
6-MB-cAMP	1.64		0.4%			
6-Bnz-cAMP	1.9		0.6%			
8-pCPT-cGMP	2.52	19.6%		240%	30%	0.008%
8-pCPT-cAMP	2.65	22.0%	0.05%			
8-Br-PET-cGMP	2.83	30.9%		10%	0.15%	1.6%
Rp-8-Br-PET-cGMPS	2.83			0.2%		
8-pCPT-2’-OMe-cAMP (Epac Activator)	2.94		0.03%	0.02%		
Sp-5,6-DCI-cBIMPS	2.99		<0.001%			

